# Infrared Thermography for the Detection and Characterization of Photovoltaic Defects: Comparison between Illumination and Dark Conditions

**DOI:** 10.3390/s20164395

**Published:** 2020-08-06

**Authors:** Sara Gallardo-Saavedra, Luis Hernández-Callejo, María del Carmen Alonso-García, Jesús Muñoz-Cruzado-Alba, Javier Ballestín-Fuertes

**Affiliations:** 1 Campus Universitario Duques de Soria, Universidad de Valladolid, 42004 Soria, Spain; 2 Centro de Investigaciones Energéticas, Medioambientales y Tecnológicas (CIEMAT), Photovoltaic Solar Energy Unit, Energy Department, 28040, Madrid, Spain; carmen.alonso@ciemat.es; 3 Fundación CIRCE, Parque Empresarial Dinamiza, Avenida Ranillas Edificio 3D, 1ª Planta, 50018 Zaragoza, Spain; jmunoz@fcirce.es (J.M.-C.-A.); jballestin@fcirce.es (J.B.-F.)

**Keywords:** infrared thermography, characterization of photovoltaic defects, bidirectional inverter

## Abstract

Newly installed renewable power capacity has been increasing incredibly in recent years. For example, in 2018, 181 GW were installed worldwide. In this scenario, in which photovoltaic (PV) energy plays a leading role, it is essential for main players involved in PV plants to be able to identify the failure modes in PV modules in order to reduce investment risk, to focus their maintenance efforts on preventing those failures and to improve longevity and performance of PV plants. Among the different systems for defects detection, conventional infrared thermography (IRT) is the fastest and least expensive technique. It can be applied in illumination and in dark conditions, both indoor and outdoor. These two methods can provide complementary results for the same kind of defects, which is analyzed and characterized in this research. Novel investigation in PV systems propose the use of a power inverter with bidirectional power flow capability for PV plants maintenance, which extremely facilitates the electroluminescence (EL) inspections, as well as the outdoor IRT in the fourth quadrant.

## 1. Introduction

Renewable energies are nowadays a completely mainstream element in the global electricity mix. Combined with energy efficiency, renewables are playing a decisive role in decreasing emissions in the energy sector and in end-use sectors. Renewable electricity has expanded, due to both transferable and safe technologies and practical policy frameworks. With 181 GW added, the newly connected renewable power capacity set new records worldwide in 2018, raising the total by more than 8% relative to 2017, leaded by solar photovoltaic (PV), with 100 GW of new solar PV capacity installed in 2018, adding a total fixed PV solar capacity of 505 GW [1,2]. In this scenario in which PV energy plays a leading role, failure recognition systems in photovoltaic plants are important to all actors involved in them. [3]. Ensuring the reliability of photovoltaic plants, as well as their durability, has been essential in recent decades. This is important to know the origin of the failures, as well as their traceability [4].

It is widely known that the cost of purchasing PV solar modules has drastically fallen in recent decades, and especially in recent years [5,6,7], which makes the idea of installing PV solar plants even more attractive. Almost all the efforts of the PV exploitation are focusing on carrying out inspections of their plants (maintenance) that allow them to maintain their efficiency (operation), with the clear objective of obtaining high productivity levels. Therefore, the idea of advanced operation and maintenance (O & M) is very attractive for the PV exploitation sector, since it will guarantee certain levels of efficiency in their PV plants [8,9].

Diverse inspection techniques have been traditionally used to detect faulty PV modules and cells. Visual inspection is one of the quickest methods to locate PV anomalies in a module [10], although only some types of faults are detected (bubbles, delamination, yellowing, browning, broken cells, oxidized or burned cells, corrosion or exposed electrical parts). The above can be completed with a laboratory analysis under a microscope [11]. By means of electrical characterization, an exhaustive analysis of the characteristics of the localized defects must be carried out. The best information from a photovoltaic module can be obtained using the current-voltage curve (I-V) [12], and its main parameters. The main problem with this technique is that it is not possible to locate the defect exactly [13]. The I-V curve provides vital information on the faults in the photovoltaic modules, and it is possible to observe degradation, cracks, problems of inappropriate resistance, anomalous operation of diodes and shadows [11]. An interesting technique, since it is applied with the photovoltaic plant in operation, is infrared thermography (IRT), for detecting heat distribution in the evaluated area. By IRT, areas or points with higher or lower heat emissivity are set, which could suggest the presence of a fault. This fact combined with the requirement of minimal instrumentation and being a non-contact practice make IRT very attractive, in addition to the great advantage of being able to be performed with unmanned aerial vehicles (UAV). A 97% average increasing in inspection efficiency between aerial and to manual inspection time is indicated in [14], comparing, i.e., 1.07 EUR/kWp reduction. Numerous works have demonstrated the validity of thermographic methods for detecting cell failure in the module space [15,16,17]. Concluding the inspection systems, electroluminescence (EL) imaging is another technique for failure identification in PV modules in which defective areas are darker, because disconnected parts do not irradiate [18]. Although it is one of the most promising maintenance techniques, its high cost has prevented its use regularly up to date [19]. It is a well-established non-destructive technology that can be used in the manufacturing process of the modules, transported to a lab after unmounting the PV modules from the site or on site, with an assembly or a specific tripod or also by means of EL cameras mounted on UAVs. This non-invasive technique allows light emission to be detected from the solar cell to the observer, and the intensity of light emission is used as an indicator of the state of the cell [20,21,22]. Based on the EL image obtained, it is possible to have a diagnosis of the state of the solar cell. Some works have shown the complementarity between the EL and TIR technique, so both methods are interesting to perform [22,23].

Regarding the sensors used for each of the inspections introduced, red, green, blue (RGB) sensors are used for visual inspections, although it can also be performed with human’s natural eye under natural sunlight [24]. In recent times, the IR senor is included together with an RGB sensor, so the exact location of the defect in the photovoltaic module is easier. Normally, IR sensor works in a spectral range between 3 μm and 14 μm, covering temperatures between −20 to 350 °C [25]. On the other hand, the peak of the EL spectrum of Si-PVs is at 1.15 μm, and it is more often reported at 1.1–1.2 μm. The spectral response range of short wave infrared (SWIR) cameras is around 0.9–1.7 μm [26]. Therefore, it is possible to say that it is very suitable for detecting problems in silicon cells.

Among the different systems for defects detection, conventional IRT is one of the fastest and least expensive techniques. Although it has been traditionally applied only with the PV plant in operation, which has the great advantage of not losing energy, it can also be applied while injecting current to the modules in the fourth quadrant, simultaneously or subsequent to EL inspections. Some authors term these two kinds of thermographic inspections as outdoor (illuminated) and indoor (dark) conditions, respectively [10,27,28,29,30]. However, novel research in PV systems propose the use of a power inverter with bidirectional power flow capability for utility-scale PV plants maintenance, using the EL technique, together with the flexibility provided by this type of devices [19]. In this case, it allows and facilitates the performance of outdoor IRT inspection injecting current simultaneously or subsequent to EL, complementing the results. We propose to denominate these inspections as outdoor (dark) IRT or outdoor (illuminated) IRT in the fourth quadrant along this paper. Outdoor (dark) and outdoor (illuminated) IRT provide complementary results for the same kind of defects, which are analyzed and characterized in this research. If this power inverter with bidirectional power flow capability was not available, the modules should be disconnected from the inverter and connected to a power source to do the inspections. Therefore, it is a novel system in PV plants which extremely facilitates the EL inspection as well as the outdoor (dark) IRT inspection, as it will be proved in this paper. Indoor IRT can be done in operation of the modules if proper lamps [31] in a solar simulator are used and injecting current in forward direction to the terminals using a current source, which can be denominated indoor (illuminated). However, this inspection technique will not be used along this paper, as it is not the purpose of the study. The introduced terms should not be confused with which other authors term as indoor photovoltaics (IPVs) [31], indoor lighting conditions [32] or indoor solar cells [33], which implies the conversion of the indoor light energy into direct electricity.

The main objective of this research is to analyze the outdoor (dark) IRT inspection using a power inverter with bidirectional power flow capability as a new inspection technique in PV plants and to demonstrate the things in common and the differences between outdoor (illuminated) and outdoor (dark) IRT relating both with other inspection techniques, as I-V curves and EL. The paper begins with the introduction to the topic covered, followed by the section on materials and methods, which explains the operating points of a PV system based on its voltage and current and details the sensors, equipment and modules used. Below are the main results of the study, which lead to its analysis and discussion. Finally, the conclusions of the work are indicated.

## 2. Materials and Methods

### 2.1. I-V Curve and Its Quadrants

**Figure 1:**
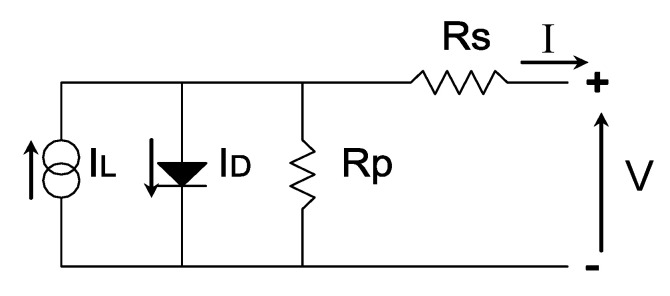
One-diode model of a photovoltaic (PV) cell including the series and shunt resistances.

**Figure 2:**
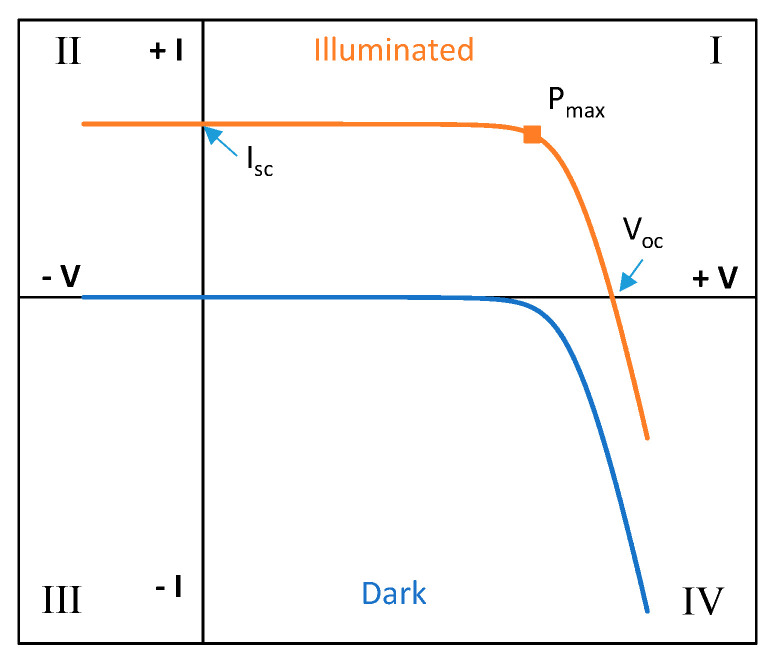
Illuminated and dark I-V curve of a PV device. Characteristic points are shown.

The working principle of a solar PV cell consists in absorbing the light and convert it into electricity. The I-V curve under illumination represents all the possible combination of current and voltage of the PV device under certain conditions of irradiance and temperatures. It represents the superposition of the dark diode current with the photogenerated current, due to illumination, and can be described with the well-known one exponential model, including the series and shunt resistances [34], as shown in Figure 1. Equation (1) shows this expression:

(1) $$I=\;I_{L}-I_{0}\left[\exp\left(\frac{V+I\;R_{s}}{m\;v_{t}}\right)-1\right]-\left(\frac{V+I\;R_{s}}{R_{sh}}\right)$$

where *I_L_* is the photogenerated current, *I_0_* is the diode saturation current, m is the diode ideality factor, *Rs* is the series resistance, *Rsh* is the shunt resistance, and *v_t_* is the thermal voltage (*K T/e*) with *K* as the Boltzmann constant, *e* as the electron charge, and *T* as the temperature in Kelvin. Figure 2 shows the typical I-V curve of a PV device both under illumination and in dark conditions, with their characteristic points in illumination: short circuit current, *Isc*, open circuit voltage *Voc*, and maximum power point *Pmax*, with its corresponding voltage *Vmax*, and current *Imax*. The criteria that has been followed in Figure 2 and in the rest of the paper is considering that when the PV device is generating current, both its current and voltage are positive (first quadrant).

The optimum operational point of a PV device is the maximum power point, which is the point searched by the PV inverters in power plants. Nevertheless, under certain conditions it is possible that some devices move to second quadrant, or operate in the fourth quadrant (dark I-V curve) for testing purposes.

**Figure 3:**
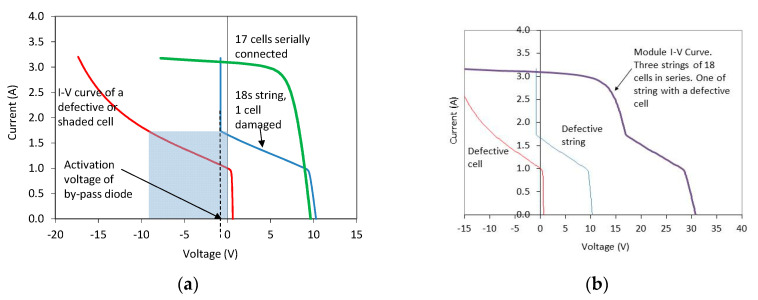
Working principle of the bypass diode. (**a**) Left: a damaged cell is associated in series with other 17 cells serially connected. The string has a bypass diode. The activation point of the bypass diode is shown. (**b**) Right: I-V curve of a PV module with three strings of 18 cells serially connected (18 s). One of the strings has a defective cell.

Operation in the second quadrant usually happens in PV modules with defective cells or in case of partial shading. A PV module is an association of PV cells, typically serially connected. If all the module cells are identical and connected in series, under maximum power point conditions the working current will be the cell *Imax* current, and the module *Vmax* will be the sum of each cell voltage. If all the cells in the module are not identical, due to differences in production, defects or shading, then the defective cell with lower current will move its operation point to the second quadrant, trying to reach the string current. In these conditions, the cell will dissipate the power produced by the other cells in the association, causing an increase in temperature. To avoid a high power dissipation which could damage the cell due to overheating [35,36], PV modules are manufactured with bypass diodes in their connection box. Bypass diodes are connected in parallel to a number of cells serially connected, and have the function of limiting the number of cells that can dissipate power into a defective one and to offer an alternative path to current in case of severe defects or partial shading. The operation of the bypass diode produce a knee in the module I-V curve, whose position depends on the number and distribution of bypass diodes in the module, and the type of defects or shading [37,38]. Figure 3 illustrates the working principle of the bypass diode.

Operation in the fourth quadrant in dark conditions is applied to perform electroluminescence imaging of the PV module [29]. In this case, the module is forward biased with a power supply, to achieve a current similar to the *Isc* of the module. Other working points, as 10% of *Isc*, are commonly selected to better distinguish certain types of failures [11,19]. 

### 2.2. Sensors

Indoor and outdoor tests have been conducted in the School of Forestry, Agronomic and Bioenergy Industry Engineering (EIFAB) of University of Valladolid, in Soria, Spain. 

**Figure 4:**
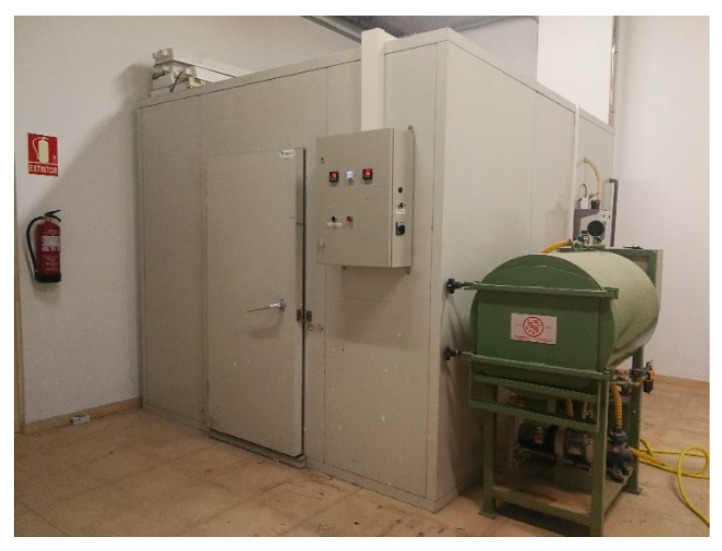
Temperature and humidity-controlled chamber at the School of Forestry, Agronomic and Bioenergy Industry Engineering (EIFAB) in Soria, Spain.

Indoor tests include indoor (dark) EL, which has been performed in controlled ambient conditions simultaneously than indoor (dark) IRT in the fourth quadrant. For these two tests, the University has a temperature and humidity-controlled chamber, which is shown in Figure 4.

EL images have been captured with a pco.1300 camera with an exposure of 5000 ms and indoor IRT images with a Flir C2 system, with a 80 × 60 pixels resolution, a thermal sensitivity of 0.1 °C and an accuracy of ±2% o ±2 °C. In EL test, when the forward voltage is connected to the cell, the internal barrier and electric field in the barrier region will decrease accordingly, which breaks the carrier balance. Then, electrons and holes radiate compound and spontaneously radiate photons outward [26]. In this case, the forward voltage has been applied with a laboratory source, adjusting the voltage to feed the short circuit current (*Isc*) individually to each tested module.

**Figure 5:**
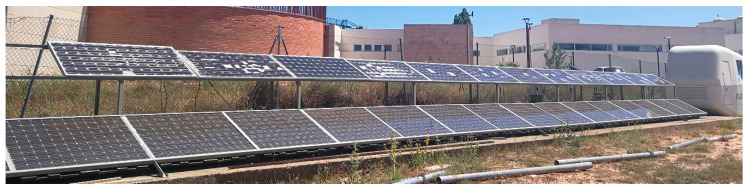
PV field of the Campus Duques de Soria of the University of Valladolid in Soria, Spain.

On the other hand, outdoor tests, including outdoor (illuminated) IRT in the first quadrant, outdoor (illuminated) IRT in the fourth quadrant and outdoor (dark) IRT have been carried out in the PV field of the Campus Duques de Soria of the University of Valladolid, which can be seen in Figure 5. The thermographic camera used outdoor is a Workswell Wiris Pro camera, with a 640 × 512 pixels resolution, a thermal sensitivity of 0.05 °C and an accuracy of ±2% o ±2 °C. This camera has a frame rate of 30 Hz, is calibrated to be used with two different lenses, 32° and 69°, and includes a Full HD RGB sensor with a resolution of 1920 × 1080 pixels and ×10 zoom. In this case, the forward voltage for tests in the fourth quadrant has been applied with a bidirectional inverter, adjusting the voltage to feed the *Isc* to the string of modules. This novel device, which is the key of the research performed as it extremely facilitates the outdoor EL inspections, as well as the outdoor (dark) IRT inspection and the outdoor (illuminated) IRT in the fourth quadrant, is in-depth detailed in the following subsection.

Solar irradiance has been taken from ATERSA 4-20mA Compen solar cell, which registers the irradiance level every minute. The ambient temperature has been taken from Advanticsys CO_2_, temperature and humidity sensor, which registers the temperature every hour. All this information is registered in the ADVANTICSYS PV plant data logger, model MPC374.

Regarding the software used for the images treatment and reporting, Imaje J [39] was used for EL images, Flir Tools [40] for indoor Flir C2 IRT images and Workswell CorePlayer [41] for the outdoor Wiris Pro IRT images.

### 2.3. Power Inverter with Bidirectional Power Flow Capability

A specific power inverter with bidirectional power flow capability was placed in the pilot-site for this study. The power inverter is a NPC I-type that has been recently developed to help in the maintenance of PV plants by means of EL image processing [19]. Indeed, the three-level neutral point clamped (NPC) configuration is one of the most extended topologies in PV inverters currently on the market. One of the main advantages of this approach is that bidirectional current flow can be useful to avoid the typical inspection procedure, in which the modules are disconnected from the inverter to be connected to a power source, saving a lot of effort and resources. 

However, PV inverters are not usually prepared to start under low-irradiance conditions, and some minor hardware modifications are required in this sense [19]. A DC-link pre-charge system is required to perform inspections in low-irradiance environments. Nevertheless, the cost of the pre-charge system is negligible compared to the cost of a utility-scale PV inverter, and the high automatization level to perform on-site inspections. In addition, the changes do not affect the inverter’s efficiency, so the modifications do not introduce any additional power losses. 

Therefore, the proposed inverter is a novel system in PV plants which extremely facilitates and place value on the on-site outdoor image processing inspections, taking special interests for the following techniques: the EL inspection; the outdoor (dark) IRT inspection; and the outdoor (illuminated) IRT inspection. 

Regarding the pilot-site of this study, a power inverter with a rated output power of 3 kW has been installed in the facilities of the solar plant. On the one hand, the output of the inverter has been connected to the 400 V three-phase grid. On the other hand, the DC input side can set the PV string voltage between 330 and 550 V. Note that during the EL test execution, this voltage range is enough to allow the current control vary between 10% and 100% of *Isc*.

**Figure 6:**
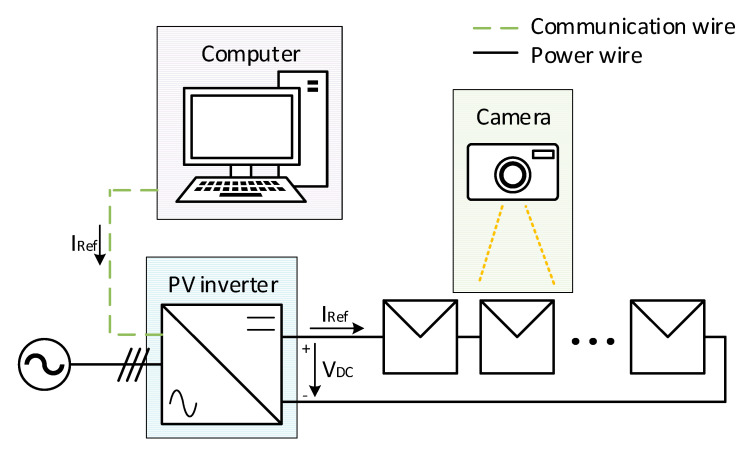
Inspection process diagram. The current setpoint ($I_{Ref}$), sent from the computer to the inverter, is controlled by means of the PV side voltage ($V_{DC}$ ).

The image capturing procedure is as follows: in standard working operation conditions, the power inverter works autonomously, taking energy from the panels and feeding it into the grid whenever there is enough solar irradiance. However, when an inspection is required, as Figure 6 shows, a computer connected to the inverter gives it the right orders. 

The user can set the desired inspection setpoint in the computer, usually the current necessary to carry out the inspection, and afterwards, proceeds to send a command to the inverter. The system, from that instant, ignores the maximum power point tracking (MPPT) algorithm, and follows the reverse reference current ($I_{Ref}$), by means of varying the DC side voltage ($V_{DC}$), until the desired current has been reached.

### 2.4. Tested Modules

**Figure 7:**
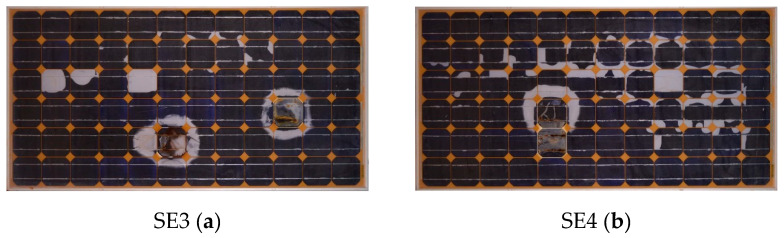

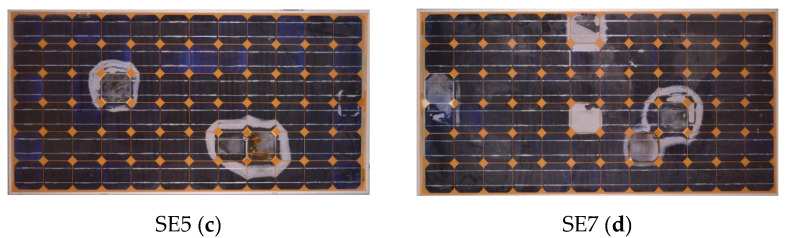
Red, green, blue (RGB) images of the 175W Eoplly mono-crystalline defective modules analyzed in the research: (**a**) module 1; (**b**) module 2; (**c**) module 3; (**d**) module 4.

**Figure 8:**
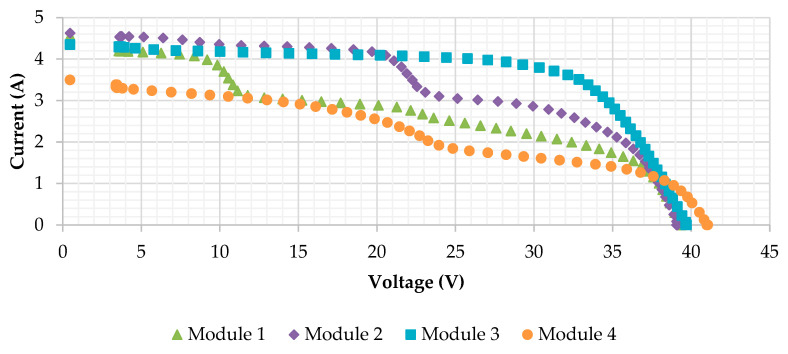
I-V curves of the 175W Eoplly mono-crystalline defective modules analyzed in the research, drawn using an I-V tracer Solar IV HT 1500V at 800, 859, 839 and 792 W/m2 for modules 1 to 4, respectively. Typical knees of the operation of the bypass diode can be observed in the I-V curves.

Four mono-crystalline modules with different kinds of defects have been tested in the present research. The main characteristics of the modules are: 72 cells (6 × 12) with 3 bypass diodes (one each 24 cells), power (P) 175W, open circuit voltage (*Voc*) 44.35 V, maximum power point (*Vmp*) 36.26 V, *Isc* 5.45 A and maximum power current (*Imax*) 4.83 A. The modules selected for the study contain several defects, in order to be able to identify failures with the different techniques, and to be able to carry out the comparison. If the modules were new and they did not show defects, the assessment would not be possible. The following Figure 7 and Figure 8 present the RGB images and the I-V curves of the analyzed modules, respectively. I-V curves have been traced using an I-V tracer Solar IV HT 1500 V at 800, 859, 839 and 792 W/m^2^ for modules 1 to 4, respectively. The interpolation to standard test conditions (STC) has not been applied, as the significant deterioration of the modules could importantly modify the coefficients of variation of *Isc* (*α*), *Voc* (*β*) and *P* (*γ*) with the temperature or other interpolation equations used by the I-V tracer, not obtaining real results. In Figure 8, typical knees of the operation of the bypass diode can be observed in the I-V curves, in which the defects induce the bypass conduction of current, as in module 1, module 2 and module 4. The defects present in module 3 do not seem to cause the operation of the bypass diodes. However, in module 1, module 2 and module 3 there is a reduction in the Voc values with respect to module 4, which could indicate the presence of short-circuited cells according to reference [11], as will be further analyzed in the discussion.

## 3. Results

This section provides a description of the results obtained in the experimental tests detailed in Materials and Methods, their interpretation, as well as the experimental conclusions that can be drawn. 

Indoor measurements have been done at 25 °C, in the temperature-controlled chamber showed in Figure 4. Outdoor and indoor IRT images irradiation, ambient temperature and module temperature are presented in Table 1. The module temperature corresponds with the average temperature of the whole surface of the module. This information will be used in the results discussion in the following section. The current level could produce quantitative variations in the temperature of the defect. However, the authors consider that there are no qualitative variations regarding the identification of the defect. 

### 3.1. Module 1

**Figure 9:**
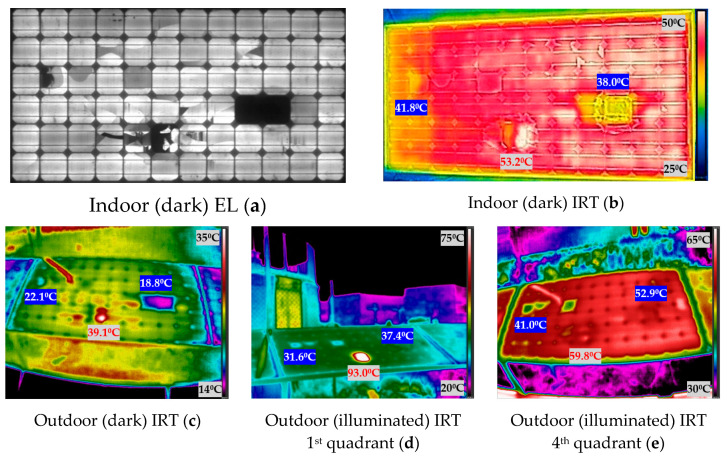
Indoor and outdoor characterization images of module 1: (**a**) indoor (dark) electroluminescence (EL); (**b**) indoor (dark) infrared thermography (IRT); (**c**) outdoor (dark) IRT; (**d**) outdoor (illuminated) IRT first quadrant; (**e**) outdoor (illuminated) IRT fourth quadrant.

Figure 9 shows the experimental results obtained when measuring the photovoltaic module 1. The Figure 9 shows the images taken indoor and outdoor, both in darkness and in illumination. In IRT images, the temperature of the spots hotter than the average temperature of the module is indicated in red, while the temperature of spots cooler than the average of the module is indicated highlighted in blue.

### 3.2. Module 2

**Figure 10:**
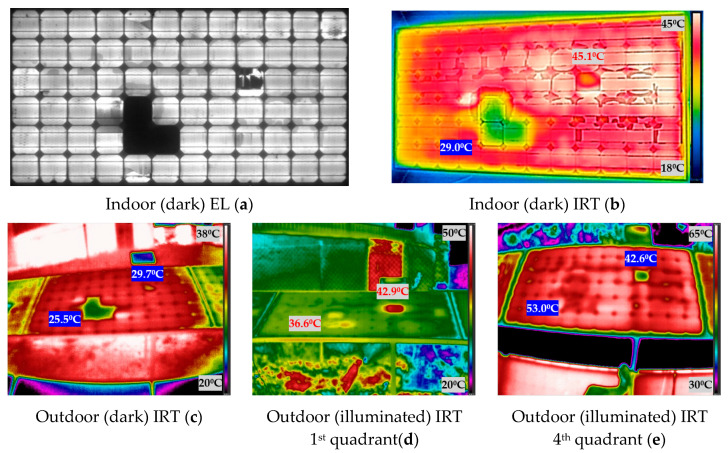
Indoor and outdoor characterization images of module 2: (**a**) indoor (dark) EL; (**b**) indoor (dark) IRT; (**c**) outdoor (dark) IRT; (**d**) outdoor (illuminated) IRT first quadrant; (**e**) outdoor (illuminated) IRT fourth quadrant.

Figure 10 shows the experimental results obtained when measuring the photovoltaic module 2. The Figure 10 shows the images taken indoor and outdoor, both in darkness and in illumination. In IRT images, the temperature of the spots hotter than the average temperature of the module is indicated in red, while the temperature of spots cooler than the average of the module is indicated highlighted in blue.

### 3.3. Module 3

**Figure 11:**
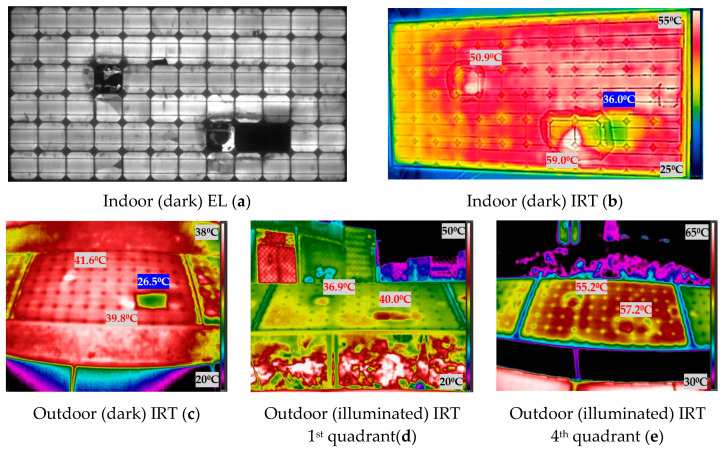
Indoor and outdoor characterization images of module 3: (**a**) indoor (dark) EL; (**b**) indoor (dark) IRT; (**c**) outdoor (dark) IRT; (**d**) outdoor (illuminated) IRT first quadrant; (**e**) outdoor (illuminated) IRT fourth quadrant.

Figure 11 shows the experimental results obtained when measuring the photovoltaic module 3. The Figure 11 shows the images taken indoor and outdoor, both in darkness and in illumination. In IRT images, the temperature of the spots hotter than the average temperature of the module is indicated in red, while the temperature of spots cooler than the average of the module are indicated highlighted in blue.

### 3.4. Module 4

**Figure 12:**
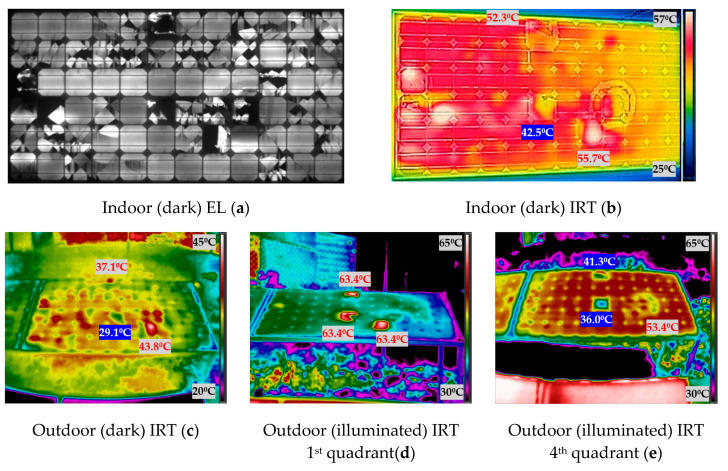
Indoor and outdoor characterization images of module 4: (**a**) indoor (dark) EL; (**b**) indoor (dark) IRT; (**c**) outdoor (dark) IRT; (**d**) outdoor (illuminated) IRT first quadrant; (**e**) outdoor (illuminated) IRT fourth quadrant.

Figure 12 shows the experimental results obtained when measuring the photovoltaic module 4. The Figure 12 shows the images taken indoor and outdoor, both in darkness and in illumination. In IRT images, the temperature of the spots hotter than the average temperature of the module is indicated in red, while the temperature of spots cooler than the average of the module is indicated highlighted in blue.

## 4. Discussion

As seen in the results section, it is possible to do IRT in different situations. However, before getting into this analysis, it is necessary for a brief reminder regarding photovoltaics. A PV module has two ways of working, in illumination and in darkness. Therefore, it is not correct to speak of IRT (or EL) just outdoor or indoor, but rather it is correct to speak of: outdoor (illuminated), outdoor (dark), indoor (illuminated), indoor (dark). Results obtained in the different situations are discussed through this section. To denominate a specific cell within a module, it will be done identifying its row with a letter and the column with a number, starting with the upper left cell of the module as cell A1.

### 4.1. Illuminated IRT

The first subject which will be discussed is the analysis of outdoor (illuminated) IRT first quadrant and outdoor (illuminated) IRT fourth quadrant. IRT in both situations is performed in illumination but the effect on the PV module is different. When the module is in illumination and receiving the injected current (fourth quadrant) (e) figures from the bidirectional inverter, the protection diodes cannot bypass the current, because they work as an open circuit with this polarity, and all cells (including damaged cells) receive all the injected current. Therefore, the damaged cells can receive all the current injected into the module. Instead, in illumination and operating normally (in the first quadrant), (d) images, the bypass diodes will protect the sub-strings with defective cells, limiting the power dissipation. In the previous figures, from Figure 9 to Figure 12, this slight difference in IRT can be seen in (d) and (e).

The operation of a PV module in the fourth quadrant under illumination is a condition that would be difficult to achieve in the field. Nevertheless, with the aid of the bidirectional inverter and for testing purposes, the operation point of the modules was moved to this quadrant to investigate the differences in temperature with respect to the other conditions. As future work, it is proposed to apply all these techniques in other plants. This research group collaborates with a company that has inverters of these characteristics in a real plant in operation. However, the objective of this article is the comparison of the different IRT techniques (illumination and darkness). It was observed that in some of the cases, for instance, in Figure 11d,e, the faulty cells increase their temperature over the module 3 average temperature, due to power dissipation in both conditions, outdoor illuminated first and fourth quadrant. In Figure 11d, the difference of temperature between the two hotspots and the module average temperature is 2.31 °C and 5.41 °C, for the left and right hotspots respectively. In Figure 11e, these differences are 4.33 °C and 6.33 °C, respectively. This can be due to the difference in the irradiation conditions between both cases, as image in Figure 11d was taken with 680 W/m^2^ and (e) with 917 W/m^2^. However, regarding Figure 12d,e, there is agreement among the spotted cells, however, they appear hotter than the module 4 average temperature in outdoor (illuminated) IRT in the first quadrant (d), and one hotter and the rest cooler in outdoor (illuminated) IRT in the fourth quadrant (e). A similar effect is found in module 2 (Figure 10).

### 4.2. Dark IRT

Secondly, with respect to the dark IRT, the technique can be performed both indoor (dark) and outdoor (dark), as can be seen in images from Figure 9 to Figure 12b,c in Section 3, respectively. Both images are similar, and complementary to the image obtained in EL (a). This means that dark IRT can be used to identify faults in PV modules similar to those identified with the EL technique, as it will be further discussed in Section 4.4. As seen in the previous Figure 9, Figure 10, Figure 11 and Figure 12, cold-spot failures can be identified, which have been defined by authors in [11], and are recognized as black cells in EL images (a). This fact can be observed in Figure 9a, which has two contiguous black cells, detected as cold spots in Figure 9b,c. Additionally, module 1 presents two cells with some inactive areas (C2 and E6) in EL (Figure 9a). One of those cells (cell C2) is detected as a cold spot, while the other one, cell E6, is shown as a hotspot in Figure 9b,c. The difference of temperature of the inactive cells (D9 and D10) with respect to the average of the module is −8.80 °C in indoor (dark) IRT (b) and −7.82 °C in outdoor (dark) IRT (b). The differences of temperature of the broken cells (C2 and E6) with respect to the average of the module are −5.0 °C and +6.4 °C, respectively, in indoor (dark) IRT (b), and −4.52 °C and 12.48 °C, respectively, in outdoor (dark) IRT (b). As seen, similar results are obtained in indoor and outdoor (dark) IRT.

In all the modules, outdoor (dark) injection of current to the PV modules has been possible thanks to a bidirectional inverter. This new device allows EL and IRT to be done in the dark, without the need to disconnect the PV modules, and without having to connect them to an external power source.

### 4.3. Comparison between Illuminated and Dark IRT

The working point in an illumination curve is different from the working point in dark conditions (in the dark curve), which is responsible of the different results between the illumination and dark IRT. This can be seen comparing Figure 9 to Figure 12 in Section 3 (b), (c) versus (d) and (e). As can be seen in the figures in Section 3, when comparing images in darkness (b) and (c) and images in illumination (d) and (e), the results are different. In dark images, the cold spots of the PV module are easily recognizable, as seen with the inactive cells in Section 4.2, while in the illuminated images, these spots do not appear, and hot spots are located. This effect can be seen in Figure 10b,c, in which the three inactive cells (D5, E5 and E6) are seen as cold spots in indoor and outdoor (dark) IRT. On the other hand, in the illuminated IRT images, Figure 10d,e, these cold spots are difficult to identify, while hot spot of damaged cell C9 is easily located. The images in darkness are equal comparing the images taken indoors and outdoors (b) and (c), respectively, since, in both cases, we work on the darkness curve as already detailed in Section 4.2. They could present some minor differences, since those outdoors could be affected by weather conditions (mainly temperature), which are introduced in Table 1.

### 4.4. Indoor (Dark) IRT and EL

It can be highlighted the similarity between the outdoor (dark) IRT images (equally for those indoors) with EL images. It is possible to affirm that outdoor (dark) IRT images are capable of detecting failures inherent in cells, and that they only appear in EL and do not appear in IRT in illumination, as seen in Section 4.3. Finally, it can be reviewed the cold-spots temperatures in (dark) IRT of completely inactive areas in the modules evaluated (cells D9 and D10 in module 1 (Figure 9a), cells D5, E5 and E6 in module 2 (Figure 10a) and cells E9 and E10 in module 3 (Figure 11a), which could correspond with short-circuited cells according to reference [11]. For the temperatures comparison, will be used the indoor (dark) IRT information (b), as it has been captured simultaneously to EL images (a). The difference of temperatures of the inactive cells with respect to the average of the module are: −8.80 °C in module 1 Figure 9b, −11.9 °C in module 2 Figure 10b, and −9.9 °C in module 3 Figure 11b. Therefore, the cooling is similar in the three cold-spot evaluated.

Consequently, it is possible to conclude that the outdoor (dark) IRT images are similar to the indoor (dark) IRT images, and highly coincide with the information seen in EL images. In contrast, outdoor (dark) IRT images are different from outdoor (illuminated) IRT images.

## 5. Conclusions

In recent years, the EL technique is gaining much interest in the scientific world, and its application in installed plants is beginning to be carried out. Traditionally, the EL technique requires the disconnection of the PV module and its connection to an external source, or the supply of a complete string through the power supply. In this work, authors have presented a bidirectional inverter that is used to carry out EL without disconnection of modules. Taking advantage of this device, authors of this work have raised the possibility of doing outdoor (dark) IRT, as a complement to EL images. The injection of current from the bidirectional inverter to the PV string will allow EL and IRT to be carried out in the dark, without the need to disconnect the installed modules.

With respect to IRT, the work has served to present the differences and similarities of said technique according to its point of operation. An outstanding conclusion is that IRT in the dark produces similar results, whether done outdoors or indoors, and furthermore, its results provide information that is significantly similar to that obtained with EL. In this sense, the detection of cold spots is very accurate, and the results are easily recoverable.

On the other hand, the work has shown that the information obtained with IRT in darkness is very different from that obtained with IRT in illumination. Therefore, the work invites to perform IRT in darkness when outdoor (dark) EL is performed, in order to have complementary information to that available with IRT in illumination.

The authors will carry out these described techniques in production plants, but these plants must have a photovoltaic inverter like the one presented. The authors will also work on advanced measurements in photovoltaic plants, which complement the conclusions obtained through images (EL and IRT).

## Figures and Tables

**Table 1:** Outdoor irradiation, ambient temperature and module temperature of the measurements in modules 1 to 4.

		Indoor (Dark) IRT (b)	Outdoor (Dark) IRT (c)	Outdoor (Illum) IRT 1st Quadrant (d)	Outdoor (Illum) IRT 4th Quadrant (e)
Module 1	W (W/m^2^)	-	-	627	927
	Tamb (°C)	25	18.2	20.7	21.6
	Tmodule (°C)	46.8	26.62	38.74	55.64
Module 2	W (W/m^2^)	-	-	657	961
	Tamb (°C)	25	18.0	20.8	21.6
	Tmodule (°C)	40.9	32.89	32.48	57.63
Module 3	W (W/m^2^)	-	-	680	917
	Tamb (°C)	25	17.6	20.9	21.8
	Tmodule (°C)	45.9	34.57	34.59	50.87
Module 4	W (W/m^2^)	-	-	545	909
	Tamb (°C)	25	17.4	20.9	21.8
	Tmodule (°C)	47.1	32.79	40.52	50.73

## References

[1] REN21 (2019). Renewables 2019 Global Status Report.

[2] Sampaio P.G.V., González M.O.A. (2017). Photovoltaic solar energy: Conceptual framework. Renew. Sustain. Energy Rev..

[3] Gallardo-Saavedra S., Hernández-Callejo L., Duque-Pérez O. (2019). Quantitative failure rates and modes analysis in photovoltaic plants. Energy.

[4] Papargyri L., Theristis M., Kubicek B., Krametz T., Mayr C., Papanastasiou P., Georghiou G.E. (2020). Modelling and experimental investigations of microcracks in crystalline silicon photovoltaics: A review. Renew. Energy.

[5] Harder E., Gibson J.M. (2011). The costs and benefits of large-scale solar photovoltaic power production in Abu Dhabi, United Arab Emirates. Renew. Energy.

[6] de La Tour A., Glachant M., Ménière Y. (2013). Predicting the costs of photovoltaic solar modules in 2020 using experience curve models. Energy.

[7] Raugei M., Frankl P. (2009). Life cycle impacts and costs of photovoltaic systems: Current state of the art and future outlooks. Energy.

[8] Spertino F., Corona F. (2013). Monitoring and checking of performance in photovoltaic plants: A tool for design, installation and maintenance of grid-connected systems. Renew. Energy.

[9] Moore L.M., Post H.N. (2008). Five years of operating experience at a large, utility-scale photovoltaic generating plant. Prog. Photovolt. Res. Appl..

[10] Köntges M., Kurtz S., Packard C., Jahn U., Berger K.A., Kato K., Friesen T., Liu H., Van Iseghem M. (2014). Review on Failures of Photovoltaic Modules.

[11] Gallardo-Saavedra S., Hernández-Callejo L., del Carmen Alonso-García M., Santos J.D., Morales-Aragonés J.I., Alonso-Gómez V., Moretón-Fernández Á., González-Rebollo M.Á., Martínez-Sacristán O. (2020). Nondestructive characterization of solar PV cells defects by means of electroluminescence, infrared thermography, I-V curves and visual tests: Experimental study and comparison. Energy.

[12] Fernández E.F., Montes-Romero J., de la Casa J., Rodrigo P., Almonacid F. (2016). Comparative study of methods for the extraction of concentrator photovoltaic module parameters. Sol. Energy.

[13] Schuss C., Remes K., Leppanen K., Saarela J., Fabritius T., Eichberger B., Rahkonen T. (2018). Detecting Defects in Photovoltaic Panels with the Help of Synchronized Thermography. IEEE Trans. Instrum. Meas..

[14] Huerta Herraiz Á., Pliego Marugán A., García Márquez F.P. (2020). Photovoltaic plant condition monitoring using thermal images analysis by convolutional neural network-based structure. Renew. Energy.

[15] Tsanakas J.A., Ha L., Buerhop C. (2016). Faults and infrared thermographic diagnosis in operating c-Si photovoltaic modules: A review of research and future challenges. Renew. Sustain. Energy Rev..

[16] Besold S., Hoyer U., Bachmann J., Swonke T., Schilinsky P., Steim R., Brabec C.J. (2014). Quantitative imaging of shunts in organic photovoltaic modules using lock-in thermography. Sol. Energy Mater. Sol. Cells.

[17] Breitenstein O., Langenkamp M., Lang O., Schirrmacher A. (2001). Shunts due to laser scribing of solar cells evaluated by highly sensitive lock-in thermography. Sol. Energy Mater. Sol. Cells.

[18] Du B., Yang R., He Y., Wang F., Huang S. (2017). Nondestructive inspection, testing and evaluation for Si-based, thin film and multi-junction solar cells: An overview. Renew. Sustain. Energy Rev..

[19] Ballestín-Fuertes J., Muñoz-Cruzado-Alba J., Sanz-Osorio J.F., Hernández-Callejo L., Alonso-Gómez V., Morales-Aragones I., Gallardo-Saavedra S., Martínez-Sacristan O., Moretón-Fernández Á. (2020). Novel Utility-Scale Photovoltaic Plant Electroluminescence Maintenance Technique by Means of Bidirectional Power Inverter Controller. Appl. Sci..

[20] Hallam B., Hamer P., Kim M., Nampalli N., Gorman N., Chen D., Chan C., Abbott M., Wenham S. Field Inspection of PV Modules: Quantitative Determination of Performance Loss due to Cell Cracks using EL Images. Proceedings of the 2017 IEEE 44th Photovoltaic Specialist Conference (PVSC).

[21] Jahn U., Herz M., Köntges M., Parlevliet D., Paggi M., Tsanakas I., Stein J.S., Berger K.A., Ranta S., French R.H. (2018). Review on Infrared and Electroluminescence Imaging for PV Field Applications.

[22] Deitsch S., Christlein V., Berger S., Buerhop-Lutz C., Maier A., Gallwitz F., Riess C. (2019). Automatic classification of defective photovoltaic module cells in electroluminescence images. Sol. Energy.

[23] Ebner R., Zamini S., Újvári G. Defect analysis in different photovoltaic modules using electroluminescence (EL) and infrared (IR)-thermography. Proceedings of the 25th European Photovoltaic Solar Energy Conference and Exhibition/5th World Conference on Photovoltaic Energy Conversion.

[24] Santhakumari M., Sagar N. (2019). A review of the environmental factors degrading the performance of silicon wafer-based photovoltaic modules: Failure detection methods and essential mitigation techniques. Renew. Sustain. Energy Rev..

[25] Gallardo-Saavedra S., Hernández-Callejo L., Duque-Perez O. (2018). Technological review of the instrumentation used in aerial thermographic inspection of photovoltaic plants. Renew. Sustain. Energy Rev..

[26] Yang R., Du B., Duan P., He Y., Wang H., He Y., Zhang K. (2020). Electromagnetic Induction Heating and Image Fusion of Silicon Photovoltaic Cell Electrothermography and Electroluminescence. IEEE Trans. Ind. Informatics.

[27] Waqar Akram M., Li G., Jin Y., Chen X., Zhu C., Zhao X., Aleem M., Ahmad A. (2019). Improved outdoor thermography and processing of infrared images for defect detection in PV modules. Sol. Energy.

[28] Ebner R., Kubicek B., Ujvari G., Novalin S., Rennhofer M., Halwachs M. (2015). Optical characterization of different thin film module technologies. Int. J. Photoenergy.

[29] Berardone I., Lopez Garcia J., Paggi M. Quantitative analysis of electroluminescence and infrared thermal images for aged monocrystalline silicon photovoltaic modules. Proceedings of the 2017 IEEE 44th Photovoltaic Specialist Conference (PVSC).

[30] Gallardo S., Moretón A., Jiménez M.M., Alonso V., Hernández L., Morales J.I., Martínez O., González M.A., Jiménez J. Failure diagnosis on photovoltaic modules using thermography, electroluminescence, RGB and I-V techniques. Proceedings of the 36th European Photovoltaic Solar Energy Conference and Exhibition (PVSEC2019).

[31] Venkateswararao A., Ho J.K.W., So S.K., Liu S.W., Wong K.T. (2020). Device characteristics and material developments of indoor photovoltaic devices. Mater. Sci. Eng. R Rep..

[32] Goo J.S., Shin S.C., You Y.J., Shim J.W. (2018). Polymer surface modification to optimize inverted organic photovoltaic devices under indoor light conditions. Sol. Energy Mater. Sol. Cells.

[33] Vincent P., Shin S.C., Goo J.S., You Y.J., Cho B., Lee S., Lee D.W., Kwon S.R., Chung K.B., Lee J.J. (2018). Indoor-type photovoltaics with organic solar cells through optimal design. Dye. Pigment..

[34] Wolf M., Rauschenbach H. (1963). Series resistance effects on solar cell measurements. Adv. Energy Convers..

[35] Herrmann W., Wiesner W., Vaassen W. Hot spot investigations on PV modules new concepts for a test standard and consequences for module design with respect to bypass diodes. Proceedings of the Conference Record of the IEEE Photovoltaic Specialists Conference.

[36] Alonso-García M.C., Herrmann W., Böhmer W., Proisy B. (2003). Thermal and electrical effects caused by outdoor hot-spot testing in associations of photovoltaic cells. Prog. Photovolt. Res. Appl..

[37] Alonso-García M.C., Ruíz J., Chenlo F. (2006). Experimental study of mismatch and shading effects in the characteristic of a photovoltaic module. Sol. Energy Mater. Sol. Cells.

[38] Alonso-García M.C., Ruiz J.M., Herrmann W. (2006). Computer simulation of shading effects in photovoltaic arrays. Renew. Energy.

[39] (2020). Image J: Image Processing and Analysis in Java.

[40] FLIR Tools.

[41] (2020). Workswell Infrared Cameras and Systems Workswell CorePlayer.

